# Genotype and phenotype of Vietnamese patients with androgen insensitivity syndrome

**DOI:** 10.1186/1687-9856-2013-S1-P194

**Published:** 2013-10-03

**Authors:** Vu Chi Dung, Maki Fukami, Can Thi Bich Ngoc, Bui Phuong Thao, Nguyen Ngoc Khanh, Nguyen Thi Hoan, Nguyen Thanh Liem, Ngo Diem Ngoc, Nguyen Thi Phuong Mai, Pham Thu Nga, Nguyen Phu Dat, Tsutomu Ogata

**Affiliations:** 1 Vietnam National Hospital of Pediatrics, Hanoi, Vietnam; 2 National Research Institute for Child Health and Development, Tokyo, Japan

## 

Androgen insensitivity syndrome (AIS) is the most common specific cause of 46,XY disorder in sex development. The androgen signaling pathway is complex but so far, the only gene linked with AIS is the androgen receptor (*AR*)*.* Mutations in the *AR* are found in most subjects with complete AIS but in partial AIS, the rate has varied 28–73%, depending on the case selection. More than over 800 entries of mutations causing AIS, representing over 500 different *AR* mutations from more than 850 patients with AIS have been reported. We aim to describe clinical manifestations and to identify mutation of *AR* in Vietnamese patients with AIS.

This case series study included 12 patients from 9 unrelated families with AIS. The gonadal position and external genitalia were evaluated clinicaly and using ultrasound. The mutation analysis of *AR* was performed using PCR and direct sequencing.

The age of diagnosis was 1 to 83 years old. 8/12 cases were complete androgen insensitivity syndrome (CAIS) (female external genitalia) and 4 cases were predominantly female partial AIS phenotype. Four cases had two labial testes, six cases had inguinal testes and 2 cases had abdominal testes. Five different mutations of *AR* were identified from 7 cases of 3 unrelated families including three novel ones. The novel missense mutation p.L701F (c.2103G>T) was identified in a patient of 83 year of age. The novel missense mutation p.L705F (c.2113C>T) was identified in two sibs. The novel mutation p. W752S (c. 2256 G>T) was identified in a child with CAIS phenotype and had family history. The reported missense mutation p.V747M was identified in two sibs. The reported mutation p.V867M (c.2599 G>A) was identified in a child with female phenotype.

Our study identified three novel and two reported mutation in the *AR* gene that may provide us new insights into the molecular mechanisms of AIS. The expanded database of these mutations should benefit patients in the diagnosis and treatment of this syndrome.

